# SNPHunter: a bioinformatic software for single nucleotide polymorphism data acquisition and management

**DOI:** 10.1186/1471-2105-6-60

**Published:** 2005-03-18

**Authors:** Lin Wang, Simin Liu, Tianhua Niu, Xin Xu

**Affiliations:** 1 Program for Population Genetics, Harvard School of Public Health, Boston, MA 02115, USA; 2 Department of Epidemiology, Harvard School of Public Health, Boston, MA 02115, USA; 3 Division of Preventive Medicine, Department of Medicine, Brigham and Women's Hospital, Harvard Medical School, Boston, MA 02215, USA

## Abstract

**Background:**

Single nucleotide polymorphisms (SNPs) provide an important tool in pinpointing susceptibility genes for complex diseases and in unveiling human molecular evolution. Selection and retrieval of an optimal SNP set from publicly available databases have emerged as the foremost bottlenecks in designing large-scale linkage disequilibrium studies, particularly in case-control settings.

**Results:**

We describe the architectural structure and implementations of a novel software program, SNPHunter, which allows for both *ad hoc*-mode and batch-mode SNP search, automatic SNP filtering, and retrieval of SNP data, including physical position, function class, flanking sequences at user-defined lengths, and heterozygosity from NCBI dbSNP. The SNP data extracted from dbSNP via SNPHunter can be exported and saved in plain text format for further down-stream analyses. As an illustration, we applied SNPHunter for selecting SNPs for 10 major candidate genes for type 2 diabetes, including CAPN10, FABP4, IL6, NOS3, PPARG, TNF, UCP2, CRP, ESR1, and AR.

**Conclusion:**

SNPHunter constitutes an efficient and user-friendly tool for SNP screening, selection, and acquisition. The executable and user's manual are available at .

## Background

With the ever-increasing volume of single nucleotide polymorphisms (SNPs) deposited in publicly available databases such as National Center for Biotechnology Information (NCBI) dbSNP, laboratory geneticists are faced with the routine need of selecting an appropriate set of SNPs in both gene mapping and molecular evolution studies. The major bottleneck in the workflow for SNP-based studies has shifted away from SNP discovery toward SNP selection. Although it is beyond dispute that several web-based applications and stand-alone software packages are available for handling SNP data, including viewGene [1], Genotools SNP manager [2], SNPbox [3], SNPicker [4], and SNPper [5], these applications go off on a tangent when it comes to selecting the best SNP set because their applications focus on primer design (e.g. SNPbox and SNPicker), SNP visualization (e.g. viewGene), specific platform applications such as MassARRAY technology (e.g. Genotools SNP manager), and SNP search (e.g. SNPper). In light of the surging interest in haplotype inference [6,7] and haplotype-based association studies, the power of a linkage disequlibrium (LD) study is determined not only by the number of SNPs used, but also by the quality. Contemporary geneticists aim to maximize the statistical power in detecting a disease-susceptible locus by selecting a "best set" of closely linked SNPs given a limited (and often fixed) genotyping budget [8].

In the case when a large number of SNPs are available for a susceptibility gene of interest, genotyping all SNPs on all samples is an inefficient utilization of resources. Recently, a cost-effective two-stage method has been proposed to identify disease-susceptibility markers [9]. In stage I, a set of SNPs (*S*_1_), spaced in a predefined interval, is selected (e.g., evenly spaced every 3 to 5 Kb in and surrounding a candidate gene [10]). The genotypes of markers in *S*_1 _are then used to define LD blocks and to reconstruct haplotypes within blocks across the candidate gene locus in a representative random sample, *C*_1_, of the original source population (e.g., a multiethnic cohort of men and women [11]). In stage II, a representative set (*S*_2_; *S*_2 _⊆ *S*_1_) of htSNPs is selected on the basis of the LD characterization in the random sample of *C*_1_, and *S*_2 _is then genotyped in a much larger case-control set *C*_2 _(*C*_1 _⊂ *C*_2_), and haplotype-based association tests are performed in *C*_2_, nested in the original source population.

Both stages are critical to the success of an association study. However, it is not a trivial task to select *S*_1 _(i.e. a set of evenly spaced SNPs in and surrounding a candidate gene) because the number of available SNPs for each human gene varies dramatically (from <10 to >200) because of varying gene sizes and SNP densities. Furthermore, we certainly do not simply keep common SNPs in *S*_1 _[i.e., minor allele frequency (MAF) ≥ 5%]; missense and regulatory SNPs should still be considered to be included in *S*_1 _even if their MAFs fall below 5% [11]. Hand-picking *S*_1 _by "eyeballing" is extremely labor-intensive, time-consuming, and error-prone for candidate genomic regions with hundreds and thousands of SNPs. Furthermore, obtaining a SNP flanking sequence long enough (~200 bp), and with annotation of nearby potential SNPs, is essential for the successful design of a SNP genotyping assay. Unfortunately, the flanking sequences of many SNPs recorded in dbSNP are short (<100 bp) and without any annotation of nearby SNPs.

NCBI's dbSNP offers a comprehensive SNP searching tool [12]. However, tools are still needed to easily and efficiently locate the desired SNPs, to evaluate their annotations, and to export them in suitable formats for downstream analyses. To meet such needs, we have developed the program SNPHunter, a tool with a friendly graphical user interface (GUI) that works as a portal between the user and NCBI dbSNP [13]. The program can extract and export SNP data retrieved from dbSNP, import saved SNP data, and offers a very flexible SNP selection function with graphic illustration of SNP position, function and heterozygosity. Furthermore, it retrieves any arbitrarily-defined, user-specified length of SNP flanking sequence with annotation of all nearby SNPs.

## Architectural structure

**Figure 1:**
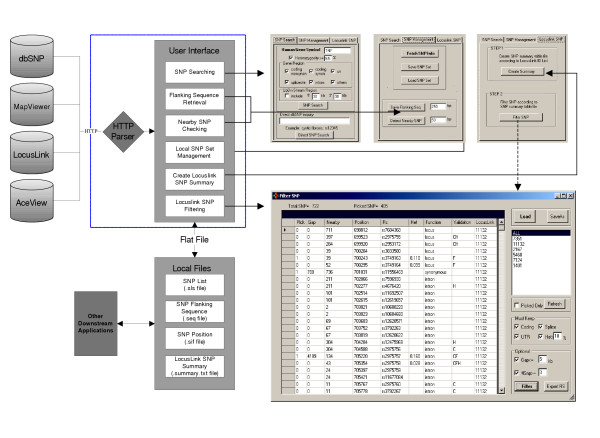
A depiction of the architecture structure of SNPHunter. SNPHunter allows the user to perform (1) an *ad hoc *search by gene symbol [an example of "Tumor Necrosis Factor" (Gene Symbol, TNF) is shown]; and (2) batch-mode search. It can be seen that a total of 722 SNPs were found on a total of 6 user-specified genes (with LocusLink ID listed on the right). With automatic filtering, 405 SNPs were picked and the gaps between them were also calculated.

SNPHunter was written using Microsoft Visual Basic .NET. A schematic diagram of the architectural framework for SNPHunter is shown in Figure 1. This tool relies on an HTTP parser that delegates the user's query to databases including dbSNP [13], MapViewer, LocusLink [14], and AceView at NCBI (Figure 1, left), and parses the retrieved data. It consists of three modular components, SNP Search, SNP Management, and LocusLink SNP. In the SNP Search module, the user inputs the gene symbol of interest and chooses SNPs based on heterozygosity (HET), chromosomal position, and functional class (Figure 1, upper right). The user can also specify whether upstream/downstream sequences of the gene should also be included for search. In the SNP Management module, the user fetches and manages detailed information for SNPs retrieved in the SNP Search module or the user's own SNP list. In the LocusLink module, SNPHunter reads in a list of LocusLink gene IDs (i.e. Entrez Gene IDs) and performs a batch-mode SNP search via LocusLink (Although NCBI LocusLink was superseded by the NCBI Entrez Gene, this SNP search mode is still fully functional).This module is very useful for obtaining SNP data with a large number of genes. SNPHunter creates a SNP summary and pops up a new "Filter SNP" panel (Figure 1, lower right). SNP filtering can be performed on all or selected genes according to user-specified filtering criteria. One advantage of the SNPHunter's local filter is that it does not rely on any Web server to perform the filtering. Once the user has downloaded all the SNP information and exported it to a local file, SNPHunter will perform filtering either automatically or manually, which means that the user can further modify the selection after automatic filtering. The selected SNP list can be exported to local directories for storage or further analyses. This batch-mode search operation is impressively fast. In the example shown in Figure 1, all the SNPs on the six genes were retrieved and downloaded in 10 sec, and automatic filtering on a regular personal computer with one Intel Pentium 4 2.8 GHz processor took another 7 sec.

## Implementation

A detailed description of the implementation of the three modules has been presented in the User's Manual [15]. In brief, since retrieval of the flanking sequences of a desired SNP relies on knowledge of its genomic coordinate, in an *ad hoc *mode, SNPHunter first pinpoints the SNP's genomic coordinate from dbSNP's reference SNP (refSNP) record, strand orientation, and the SNP's corresponding contig number. Moreover, SNPHunter communicates with the NCBI MapViewer database and retrieves the corresponding sequence centering at the desired SNP, with the sequence lengths specified by users. SNPHunter will detect all neighboring SNPs located within a user-defined radius around the SNP of interest. Once the SNP's genomic coordinate and contig data are retreived, SNPHunter also obtains nearby SNP data on all neighboring SNPs by querying dbSNP for all available SNPs that lie within the user-defined radius. Once the starting and ending coordinates of a particular gene are determined by SNPHunter through NCBI's AceView, the 5' upstream and 3' downstream regions of the gene can be retrieved according to user-defined lengths. In a batch-mode, SNPHunter communicates with NCBI's LocusLink to fetch the SNPs that reside within each LocusLink gene. Since LocusLink has a curated SNP list for each gene included in the LocusLink database, this batch-mode search offers a reliable, efficient way to conduct a systematic SNP search for a large set of candidate genes (e.g. belonging to the same biological pathway/network). Furthermore, SNP data can be stored in the user's local directories, and SNP filtering can be performed automatically according to user-defined criteria.

## Application example

To demonstrate the SNP selection process from dbSNP using SNPHunter, we applied SNPHunter for *S*_1 _selection for 10 biological candidate genes (Table 1) for a type 2 diabetes mellitus (DM) case-control study. These 10 candidate genes were chosen on the basis of their biochemical and physiological functions.

We used the following four SNP selection criteria:

(1) Genome coverage: SNPs should cover the gene region as well as its 30 Kb 5' upstream and 30 Kb 3' downstream regions (the gene sizes are shown in Table 1).

(2) Functionality priority: coding SNPs (cSNPs; including both synonymous and nonsynonymous SNPs) and splice site SNPs (ssSNPs) must be kept; for SNPs located in the 5' upstream region and 3' downstream regions, the function is defined according to existing *in vivo/in vitro *experimental data. The priority of SNP selection is nonsynonymous SNPs > synonymous SNPs > ssSNPs > 5' upstream SNPs > 3' downstream SNPs > intronic SNPs.

(3) Priority based on HET: For cSNPs and ssSNPs, no HET threshold is set (HET can be calculated using the POLYMORPHISM software [16]); for intronic and 5' upstream or 3' downstream region SNPs, those SNPs with HET values going above the threshold of 0.095 (which correspond to MAF ≥ 5%) have higher priorities.

(4) SNP density: The SNPs should be relatively evenly distributed across the gene region (as well as the 30 Kb 5' upstream and 30 Kb 3' downstream regions) with a density of 5–50 SNPs/Kb depending on the gene sizes (see Table 1). The goal is that for gene sizes < 10 Kb, we use a density of 50 SNPs/Kb; for gene sizes 10–100 Kb, we use a density of 10 SNPs/Kb; for gene sizes > 100 Kb, we use a density of 5 SNPs/Kb.

To date, there are no turn-key solutions that can select the best SNP set automatically. Our SNP selection procedure is an iterative process consisting of the following four major steps:

(a) Retrieve all SNPs regardless of HET values according to SNP selection criterion (1).

(b) Select all cSNPs and ssSNPs; in addition, 5' upstream, 3' downstream and intronic SNPs with HET ≥ 0.095 will also be selected according to SNP selection criterion (2).

(c) Enforce a relatively even SNP density according to SNP selection criterion (4). We implement this by setting the maximum inter-marker distance *d *(i.e., for a given set of selected SNPs *S*, if there exists a pair of neighboring SNPs (*SNP*_*i*_, *SNP*_*j*_), where the physical distance between *SNP*_*i *_and *SNP*_*j *_is <*d*, the program recursively picks a random SNP, say *SNP*_*k*_, between *SNP*_*i *_and *SNP*_*j *_and inserts *SNP*_*k *_in the middle of *SNP*_*i *_and *SNP*_*j*_; by mathematical induction, this process will guarantee that *S *will eventually be a saturated set, *S'*, at a resolution level of *d*). Re-adjust the marker density by iteratively adding available SNPs in the priority order set by SNP selection criterion (2) and (3) until we come to a target number of SNPs with desired density, according to SNP selection criterion (4).

(d) Include any non-redundant SNPs from sources other than dbSNP, such as from literature review.

Using these criteria and selection procedures, we selected a total of 670 SNPs for the 10 genes listed in Table 1.

**Figure 2:**
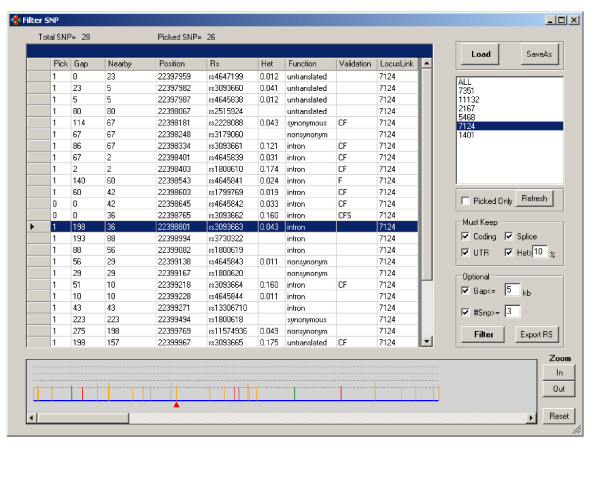
The Gene View panel which gives a graphic illustration of all the SNPs within the gene of TNF (LocusLink ID: 7124). The height of a SNP bar indicates the heterozygosity of that SNP, and the four dotted horizontal lines means heterozygosity of 0, 0.25, 0.5, and 0.75 respectively. Out of 28 available SNPs, two have been dropped and plotted as "gray". For those 26 selected SNPs, "green" means "coding: synonymy unknown" or "synonymous"; "red" means "non synonymous"; "orange" means all the others. And for the convenience of SNP selection, there is a red triangle indicating the current focused SNP, which is SNP rs3093663.

Besides SNP selection, SNPHunter allows the retrieval of genomic coordinates and flanking sequences for specific SNPs and gives graphic illustration of all the SNPs within the gene of interest as well. Figure 2 gives an illustration of the 28 SNPs found in a 2.7 Kb region spanning the tumor necrosis factor (TNF) gene from NCBI dbSNP.

## Discussion

The motivation for developing SNPHunter is to allow the efficient and accurate selection of *S*_1 _(see Background) because of its intrinsic value in LD studies, particularly in a case-control setting. A few Web resources, such as NCBI's Entrez, Ensembl's EnsMart [17] and SNPper [5] provide SNP database searching and SNP information downloading according to user-specified criteria. These tools, each with its own unique capabilities and focuses, have benefited the work of geneticists. However, few of them are dedicated solely for SNP search purposes and for the management of SNP data. Although SNPper [5] offers a very helpful function of filtering SNP sets, it is a locally stored SNP-centric database resource maintained by the Children's Hospital Informatics Program, Harvard Medical School, and requires regular data downloads from NCBI dbSNP. By contrast, SNPHunter is designed to work as a stand-alone application that retrieves the most-updated SNP and sequence data without the need for complicated local database support. Thus, the user is relieved from maintaining a local database and updating the data frequently. The ability to export and to save every dataset locally in plain text format provides the user with the freedom for later reuse or any other customized analysis without any website support. In addition, SNPHunter offers a very friendly GUI, allowing researchers without much computer background to perform SNP searches easily and efficiently. Moreover, its batch search and automatic SNP selection proved very efficient in large-scale candidate genes study. Table 2 lists features comparisons between SNPHunter and other major SNP related software/web tools.

It is worth noting that SNPHunter relies on dbSNP for data retrieval, and thus is deprived of the independence whereas other application with local database support usually has. What's more, SNP selection should not be limited to NCBI dbSNP, although dbSNP represents the largest publicly available SNP database that can be accessed via the Internet worldwide. Some SNPs reported in the earlier literature have not yet been incorporated into dbSNP. Furthermore, there are several on-going gene re-sequencing projects for selected human genes, such as SeattleSNPs or SNP500Cancer [18]. Therefore, SNPs from these other sources, if not yet included in dbSNP, should also be considered in SNP selection. Nevertheless, NCBI dbSNP has been steadily updated and has gradually emerged as one of the most comprehensive SNP depositories.

## Conclusion

In summary, SNPHunter allows for customized SNP searches (both *ad hoc*-mode and batch-mode) by directly retrieving and managing SNP information from the NCBI dbSNP database, eliminating tedious and costly local database maintenance on the user's side. To date, SNPHunter has received more than 1000 downloads worldwide. We hope this simple program can serve as an efficient and reliable tool for researchers everywhere to facilitate their genetic studies.

## Availability and requirements

Project name: SNPHunter

Project home page: 

Operating system(s): Microsoft Windows

Programming language: Visual Basic .NET

Other requirements: Microsoft .NET Framework 1.0 or above.

License: None

Any restrictions to use by non-academics: Contact authors

## Authors' contributions

Simin Liu, Tianhua Niu, and Lin Wang identified the need to develop such a program, initiated the project, and designed the basic functions. Lin Wang wrote the source code for the software and interface design. Tianhua Niu and Xin Xu contributed with ideas on overall design, feature requirements, and implementation. All authors participated in the drafting of the manuscript and approved the final version.

## Figures and Tables

**Table 1:** Size, location, and the estimated number of SNPs for each of the 10 candidate genes for type 2 diabetes mellitus.

**Gene Symbol**	**Locus-Link**	**Location**	**Size (bp) (# exons)^a^**	**Total # SNPs (#/Kb)^b^**	**# SNPs MAF ≥ 5% (density)^c^**	**Func SNPs^d^**	**# SNPs selected for S1**			
							
							**5' 30 Kb**	**3' 30 Kb**	**Gene region**	**Total**
CAPN10	11132	2q37.3	48411(32)	308 (6)	27 (0.6)	1	3	11	25	39
FABP4	2167	8q21	5616 (6)	119 (21)	4 (0.7)	2	1	1	20	22
IL6	3569	7p21	6130 (10)	291 (47)	49 (8.0)	8	3	8	40	51
NOS3	4846	7q36	24637(29)	299 (12)	67 (2.7)	28	7	6	62	75
PPARG	5468	3p25	146974(16)	627 (4)	124 (0.8)	5	0	7	142	149
TNF	7124	6p21.3	2775 (5)	381 (137)	42 (15.1)	6	17	14	20	51
UCP2	7351	11q13	34786 (26)	135 (4)	13 (0.4)	2	4	10	20	34
CRP	1401	1q21-q23	2306 (18)	171 (74)	33 (14.3)	1	5	18	21	44
ESR1	2099	6q25.1	472913 (23)	1403 (3)	207 (0.4)	9	6	16	85*	107
AR	367	Xq11.2	180270 (13)	241 (1)	15 (0.1)	8	1	1	96	98

^**a **^Gene statistics from NCBI AceView; number of exons was calculated as number of intron plus 1.

^**b **^Including SNPs belonging to all 6 NCBI dbSNP's function classes (i.e. Coding Nonsynonymous, Coding Synonymous, mRNA UTR, Splice site, Intron, and Others) that cover the gene locus as well as the 30-Kb 5' upstream and 30-Kb 3' downstream flanking sequences of the locus.

^**c **^Note that a subset of dbSNP entries do not include information on allele frequency or heterozygosity. The numbers of SNPs reported here indicate those SNPs with MAF information and MAF ≥ 5%, including 30-Kb 5' upstream and 30-Kb 3' downstream flanking regions.

^**d **^Defined as Coding Nonsynonymous, Coding Synonymous, or Splice-site SNPs.

* SNP distance (maximum inter-marker distance between selected SNPs) was controlled at 10 Kb for the ESR1 gene.

**Table 2:** Comparisons between SNPHunter and other publicly available software/tool.

**Software/Web tool**	**Type**	**Maintenance requirement**	**SNP search related functions**					**Primer design**
				
			**SNP search**	**Arbitrary flanking sequence length**	**Nearby SNP annotation**	**SNP selection interface**	**Graphic SNP illustration**	
NCBI dbSNP^**a**^	Web-Server	None for user; server contains latest updated data	Yes	No	No	No	Yes	No
SNPper	Web-Server	None for user; server needs to update periodically	Yes^**b**^	Yes	Yes	No	No	Yes
SNPHunter	Web-Client (depends on dbSNP for new SNP retrieval)	None for user; rely on dbSNP for data update	Yes^**b**^	Yes	Yes	Yes^**c**^	Yes	No
SNPicker	Web-Client (depends on NEB restriction-enzyme data)	None for user; rely on NEB for data update	No	No	No	No	No	Yes
SNPBox	Web-Server	None	No	No	No	No	No	Yes
viewGene	Software depends on annotated record	Needs to download annotated data	Yes	Yes	Yes	No	Yes	No

^**a **^dbSNP's Entrez query.

^**b **^Both SNPper and SNPHunter can do batch search for a list of genes instead of single gene at a time.

^**c **^SNPHunter offers automatic SNP selection as well as manual selection.
